# Case report: severe asymptomatic hyponatremia in Prader-Willi Syndrome

**DOI:** 10.1186/s12887-016-0563-4

**Published:** 2016-02-18

**Authors:** Daniel Landau, Harry J. Hirsch, Varda Gross-Tsur

**Affiliations:** Department of Pediatrics B, Schneider Children’s Medical Center of Israel, Petach Tikva, Israel and Faculty of Health Sciences, Ben-Gurion University, Beer Sheva, Israel; Neuropediatric Unit, Department of Pediatrics, Shaare Zedek Medical Center, P.O.B 3235, Jerusalem, 91031 Israel; The Faculty of Medicine, the Hebrew University, Jerusalem, Israel

**Keywords:** Prader-Willi syndrome (PWS), Hyponatremia, Anti-diuretic hormone (ADH), Adrenocortioctrophic hormone (ACTH) test

## Abstract

**Background:**

Prader-Willi syndrome is a complex neurogenetic, multisystem disorder. Despite the variable endocrine abnormalities and hypothalamic-pituitary axis dysfunction, hyponatremia has been reported in only a few PWS patients. In previously reported PWS individuals, hyponatremia was associated with abnormal fluid intake or during desmopressin treatment.

**Case presentation:**

We describe an infant with Prader-Willi syndrome who had severe, prolonged asymptomatic hyponatremia without a history of excessive fluid intake or desmopressin treatment. We compare the findings with those of the few other reported cases and describe, for the first time, results of a hypertonic saline infusion test and studies of adrenal cortical function.

**Conclusion:**

Hyponatremia should be suspected in children with Prader-Willi syndrome, especially in infants with severe failure to thrive. Further studies are needed to determine the pathophysiology of hyponatremia in this syndrome.

**Electronic supplementary material:**

The online version of this article (doi:10.1186/s12887-016-0563-4) contains supplementary material, which is available to authorized users.

## Background

Prader-Willi syndrome (PWS) is a complex neurogenetic, multisystem disorder with a prevalence of 1/15,000 to 1/30,000, caused by lack of expression of genes on the paternally inherited chromosome 15q11.2-q13. PWS is characterized by dysmorphic features, hypotonia, cognitive and behavioral abnormalities, impaired satiety response leading to morbid obesity, and multiple endocrine abnormalities including growth hormone deficiency, hypogonadism, impaired glucose tolerance and diabetes mellitus, and hypothyroidism [1]. Hypothalamic dysfunction has been implicated in many manifestations of the syndrome. Hyponatremia has rarely been observed despite the variable endocrine abnormalities, hypothalamic-pituitary axis dysfunction, and abnormal eating behavior. Studies of sodium homeostasis or adrenocortical function in hyponatremic PWS children or adults have not been previously described.

## Case presentation

Our patient was diagnosed with PWS at the age of 3 weeks, based on the typical neonatal characteristics including severe hypotonia, poor feeding due to inability to suck, dysmorphic features, and undescended testes. Genetic testing showed abnormal methylation and confirmed the diagnosis of PWS due to deletion of paternal genes in chromosome 15q11-q13. The child was discharged from the neonatal unit at the age of 3 weeks. At home he was very weak, had a very poor appetite, did not cry for food, took oral feeds very slowly, and had very poor weight gain. Due to recurrent high fevers up to 42 °C and an average temperature of 38–39 °C, he was hospitalized several times with a diagnosis of pyrexia of unknown origin. Treatment with antibiotics was ineffective. A sleep study at age 10 months revealed mild obstructive sleep apnea, with oxygen saturation dropping to 70 % but a hypopnea/apnea index of only two per hour. He was referred for adeno-tonsillectomy. Routine pre-operative blood tests, at age 15 months showed a serum sodium value of 118 meq/L. Sodium values, measured three and 8 months previously, were normal (135 meq/L). Parents denied use of medications or any recent diarrhea or vomiting. On admission, his temperature was 38.4 °C; otherwise his vital signs were normal. While awake, his oxygen saturation was 95 % but dropped to 82 % while asleep. No evidence of respiratory distress was seen. His weight was 9.8 kg (−0.84 SD) and length 75 cm (−1.05 SD). There were no clinical signs of dehydration or edema. His fontanelle was not sunken, mucous membranes were moist, and tears were seen on crying. He had bilateral undescended testes. Neurological exam showed head lag and generalized hypotonia. Deep tendon reflexes were difficult to elicit. Eye movements were normal. He did not crawl or sit. There were no focal neurologic signs.

A chest x-ray showed increased pulmonary markings, but nasal washes for a panel of respiratory viruses were negative. A complete blood count showed leukocytosis (18.8×10^9^/L) with a left shift (10.0×10^9^/L neutrophils), but blood cultures were negative. Serum sodium was 121 meq/L(121 mmol/l), potassium 4.8 meq/L (4.8 mmol/l), chloride 89 meq/L (89 mmol/l), uric acid 2.9 mg/dL (173 μmol/L), urea 17 mg/dL (3.03 mmol/L), creatinine 0.12 mg/dL (10.6 μmol/L), and glucose 104 mg/dL (5.77 mmol/L). Serum osmolality was appropriately low, 260 mosm/L. On urinalysis, specific gravity was 1.015, protein +2 and blood +1, but there was no proteinuria or hematuria on repeated urinalyses. Urine osmolality (324 mosm/L) was inappropriately concentrated for the state of serum hypo-osmolality. Urine sodium obtained after a single 0.9 % NaCl intravenous bolus of 20 ml/kg was appropriately elevated (80 meq/L (80 mmol/l)), suggesting lack of total body sodium deficit. Thyroid function studies showed a free thyroxine of 1.0 ng/dL (normal: 0.8–1.5) (12.9 pmol/L) and mildly elevated TSH (6.1 mIU/L, normal 0.39–4). Morning cortisol was normal (22.6 ug/dL) (623 nmol/L). Brain MRI showed mild external hydrocephalus but the hypothalamus and pituitary regions were normal.

The child did not exhibit any change in his neurological condition while hyponatremic. He was treated with supplemental oxygen, a short course of oral amoxicillin and fluid restriction. Serum sodium remained < 130 meq/L (<130 mmol/L) up to hospital day 11. No evidence of polyuria or dehydration was seen during the fluid restriction regimen adopted for therapy, thus ruling out cerebral salt wasting. At the end of this treatment the baby lost 600 g in weight and his serum sodium increased to132 meq/L (132 mmol/l).

After the hyponatremia had resolved, the baby underwent a hypertonic saline stimulation test according to the protocol of Mohn et al. [2]. Written informed consent was obtained from his parents. Plasma ADH levels rose appropriately from 1 to 3 pg/ml while serum osmolality increased from 285 to 297 mosm/L during the 1 h hypertonic saline infusion. In the past 10 months since this episode, hyponatremia has not recurred. An ACTH stimulation test was performed at age 30 months. Serum cortisol was 658 nmol/l and rose to 990 nmol/l at 60 min after injecting tetracosactide (aqueous Synacthen, Novartis Pharmaceuticals) 125 μg intravenously. The basal ACTH level was elevated, 40.5 pmol/l (normal range 1.9–10.2 pmol/l) most likely reflecting a stress response due to hypoglycemia (glucose 43 mg/dl (2.4 mmol/l)) in a young, fasting infant [3]. These results rule out primary or central adrenal insufficiency. Serum aldosterone (746 pmol/l) and renin (75.7 pg/ml (1.79 pmol/l)) were in the normal range for age. The "CARE" checklist is available as Additional file 1. This child’s clinical course is summarized in the accompanying supplemental time-line file (Additional file 2).

Food-related issues and endocrine abnormalities are typical for PWS. Although a major feature of the syndrome is severe hyperphagia due to lack of satiety, feeding difficulties and failure to thrive are usually seen in PWS infants, while weight gain begins around age 2 to 4 years [4]. Hyponatremia in PWS has been reported only in the context of excess water intake or during treatment with desmopressin [5, 6]. Although the possibility of inappropriate ADH secretion was suggested, there are no published studies describing the mechanisms responsible for hyponatremia in PWS.

The differential diagnosis of asymptomatic hyponatremia includes a spectrum of water balance disorders, and includes abnormalities of various stages of arginine-vasopressin (AVP) secretion and responsiveness [7]. Our patient had severe, refractory hyponatremia discovered during a mild respiratory illness. Simultaneous analysis of blood and urine revealed inadequate urine dilution in response to low serum osmolarity in a euvolemic child, suggestive of a transient state of inappropriate ADH secretion. Hyponatremia in PWS has rarely been described in the medical literature. Akefeldt described water intake among a cohort of PWS children and young adults [5]. He found that 76 % of PWS infants had an extremely small daily intake of water, but 15 % increased the daily water intake to unusually high amounts. The majority of individuals who increased their water consumption to extreme values belonged to the chromosome 15q-non-deletion group (as opposed to deletions), and two developed hyponatremia while receiving psychiatric medication. Given the numerous central endocrine abnormalities in this syndrome, a problem in osmotic sensing and response (“reset osmostat”) could be an etiology for hyponatremia in PWS [8]. With the increase in serum osmolality during the hypertonic saline stimulation test in our patient, ADH levels increased appropriately.

Many of the endocrine abnormalities, including growth hormone deficiency, central hypothyroidism, and central adrenal insufficiency in PWS have been ascribed to hypothalamic or pituitary dysfunction. The prevalence of central adrenal insufficiency in PWS and its role in the etiology of sudden death remain uncertain. Although serum potassium levels in our patient were normal, hyperkalemia is not a consistent presenting sign of primary adrenal insufficiency in childhood. A combination of chronic or subacute clinical symptoms, hypotension, and hyponatremia should raise suspicion of adrenal insufficiency. Nevertheless, the normal cortisol levels before and after administration of tetracosactide (Synachten) rule out adrenal insufficiency. Hyponatremia may also be due to hypothyroidism. Central hypothyroidism, with a normal thyroid stimulating hormone value and low free thyroxine level, has been documented in up to 25 % of individuals with PWS, with a mean age of diagnosis and treatment of 2 years [9]. Free thyroxine was normal in our patient and TSH was only slightly elevated. Pulmonary diseases, particularly pneumonia (viral, bacterial, tuberculous), can be associated with mild hyponatremia between 130 and 135 meq/L. The role of SIADH in pneumonia is controversial.

Hyponatremia is the most commonly occurring electrolyte disorder and is associated with increased morbidity and mortality, independent of the underlying disease, and presenting with a wide spectrum of neurological signs and symptoms, ranging from adynamia and gait disturbances, to syncope or coma. Chronic hyponatremia may be asymptomatic, however mild hyponatremia may be associated with subtle neurological abnormalities such as loss of appetite and nausea [10]. It is possible that hyponatremia contributed to some of the clinical features seen in our patient such as severe feeding problems and hypotonia. The prevalence of hyponatremia among PWS infants with severe hypotonia and failure to thrive is unknown. Routine measurement of serum electrolytes may be indicated in this population.

## Conclusions

We described an infant with PWS and severe asymptomatic hyponatremia. Unlike previous reported cases, our patient had no history of abnormal fluid intake and was not treated with desmopressin.. We reported results of a hypertonic saline stimulation test and assessment of adrenocortical function. Further studies are needed to elucidate the etiology of hyponatremia in PWS infants, to determine the true incidence of hyponatremia in these patients, and to assess the possible impact of “asymptomatic” hyponatremia on the clinical signs and symptoms.

### Consent

Written informed consent was obtained from the parents of the patient described in this study. A copy of the written consent is available for review by the Editor of this journal.

## Additional files

Additional file 1:
**CARE Checklist (2013) of information to include when writing a case report.** (DOCX 1488 kb) Additional file 2:
**Time-line summarizing the clinical course of the child described in this case report.** (PPTX 76 kb)

## Abbreviations

ACTH: Adrenocorticotropic hormone
ADH: Anti-diuretic hormone
PWS: Prader-Willi syndrome

## References

[1] Cassidy SB, Schwartz S, Miller JL, Driscoll DJ (2012). Prader-Willi syndrome. Genet Med.

[2] Mohn A, Acerini CL, Cheetham TD, Lightman SL, Dunger DB (1998). Hypertonic saline test for the investigation of posterior pituitary function. Arch Dis Child.

[3] Crofton PM, Midgley PC (2004). Cortisol and growth hormone responses to spontaneous hypoglycaemia in infants and children. Arch Dis Child.

[4] Miller JL, Lynn CH, Driscoll DC, Goldstone AP, Gold JA, Kimonis V (2011). Nutritional phases in Prader-Willi syndrome. Am J Med Genet Part A.

[5] Akefeldt A (2009). Water intake and risk of hyponatraemia in Prader-Willi syndrome. J Intellect Disabil Res.

[6] Robson WLM, Shashi V, Nagaraj S, Norgaard JP (1997). Water intoxication in a patient with the Prader-Willi syndrome treated with desmopressin for nocturnal enuresis. J Urol.

[7] Ranadive SA, Rosenthal SM (2011). Pediatric disorders of water balance. Pediatr Clin North Am.

[8] Thiagarajan R, La Gamma E, Dey S, Blethen S, Wilson TA (1996). Hyponatremia caused by a reset osmostat in a neonate with cleft lip and palate and panhypopituitarism. J Pediatr.

[9] Diene G, Mimoun E, Feigerlova E, Caula S, Molinas C, French Reference Centre for PWS (2010). Endocrine disorders in children with Prader-Willi syndrome--data from 142 children of the French database. Horm Res Paediatr.

[10] Sahay M, Sahay R (2014). Hyponatremia: a practical approach. Indian J Endocrinol Metab.

