# Supplementary material for: Case report: severe asymptomatic hyponatremia in Prader-Willi Syndrome
Source: BMC Pediatr. 2016 Feb 18;16:28. doi: 10.1186/s12887-016-0563-4 (PMC4758139; doi:10.1186/s12887-016-0563-4)
Supplement: Additional file 2: — Time-line summarizing the clinical course of the child described in this case report. (PPTX 76 kb) [file 12887_2016_563_MOESM2_ESM.pptx]

## Slide 1
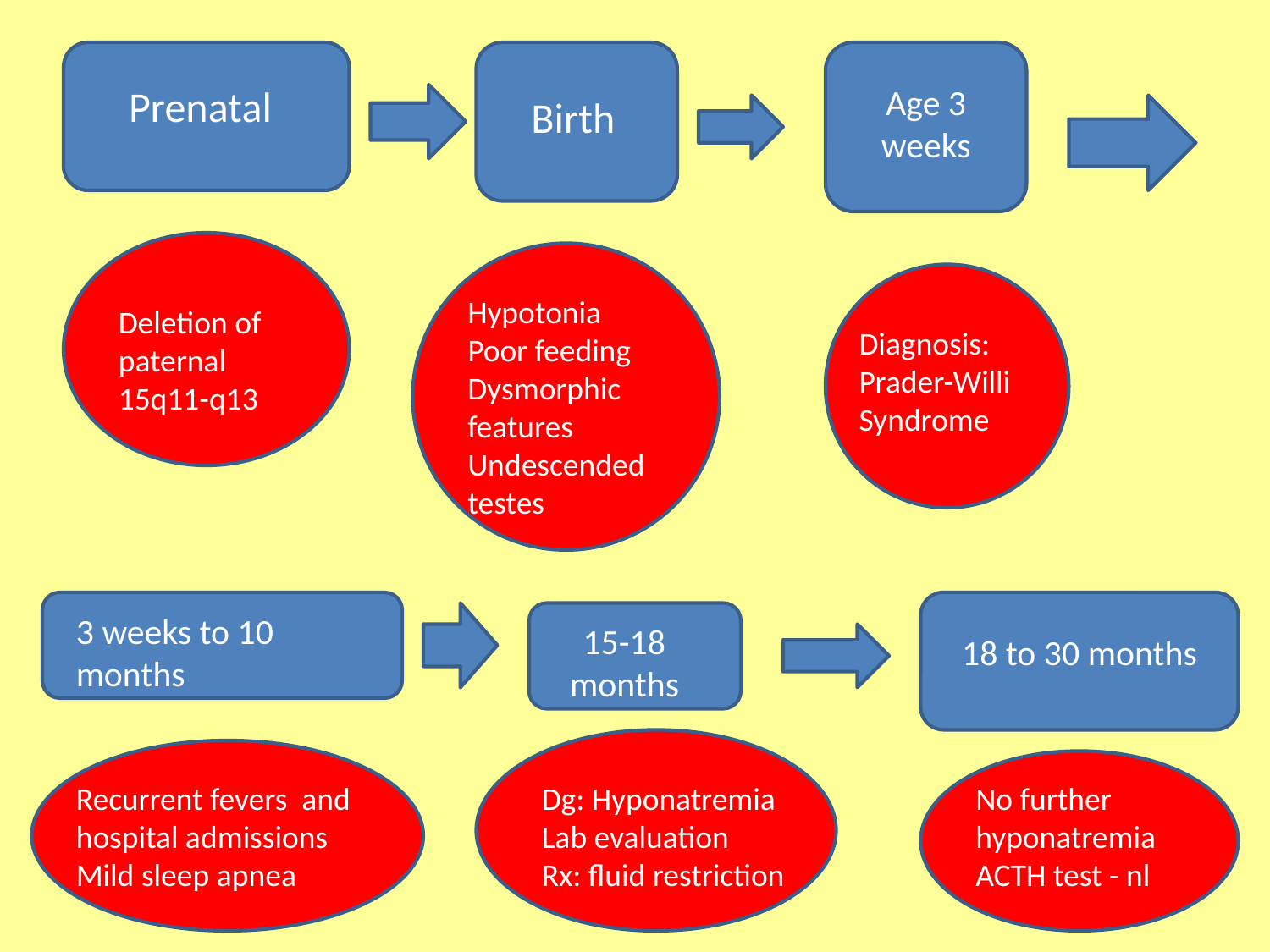

Prenatal
Age 3 weeks
Birth
Hypotonia
Poor feeding
Dysmorphic features
Undescended testes
Deletion of paternal 15q11-q13
Diagnosis:
Prader-Willi Syndrome
3 weeks to 10 months
15-18 months
18 to 30 months
Recurrent fevers and hospital admissions
Mild sleep apnea
Dg: Hyponatremia
Lab evaluation
Rx: fluid restriction
No further hyponatremia
ACTH test - nl
